# The Value of Ampullary Dilatations, Etc.

**Published:** 1888-09

**Authors:** 


### THE SEMIOLOGICAL VALUE OF AMPULLARY DILATATIONS OF THE SMALL LINGUAL VESSELS.

Dr. Gillot, of Autun, after a careful study of this subject, comes to the conclusion that ampullary or aneurismal dilatations of the sublingual capillaries occur almost exclusively in arthritic subjects, and that if all arthritics do not present them, they that have them are arthritic. Arthritism causes thinning the walls of small vessels. Whenever blood-pressure is increased, from whatever cause, cardiac hypertrophy, plethora, obstacles to respiration, etc., these aneurismal dilatations are subjected to strong tension, which may cause rupture. [But how many cases of such rupture are on record.] Every person, says Gillot, that has ampullary dilatations of the tongue may be suspected of having miliary aneurisms of the brain; and the consequences of cerebral hæmorrhage from this cause are well-known. The aspect of these miliary tumors of the tongue, their increase in size, their more or less deep color, often reveal to the physician the increased cerebral vascular tension, and should put him on his guard against threatening apoplexy. From an analysis of several hundred cases of cerebral hæmorrhage, Gillot claims that coexisting ampullary dilatations of the tongue were more developed or more numerous on the side of the cerebral hæmorrhage, and on the opposite side to the paralysis.—*L'Union Médicale*, No. 66, 1888.

